# Postural sway variability in young adults presents higher complexity during morning compared to evening hours while in older adults remains the same

**DOI:** 10.1007/s00221-025-07121-9

**Published:** 2025-06-16

**Authors:** Vasileios Mylonas, Stylianos Grigoriadis, Christos Chalitsios, Nick Stergiou, Thomas Nikodelis

**Affiliations:** 1 AUTH Biomechanics, Department of Physical Education, & Sport Science, Aristotle University, 570 01 Thessaloniki, Greece; 2 Present Address: Division of Biomechanics and Research Development, Department of Biomechanics and Center for Research in Human Movement Variability, University of Nebraska at Omaha, 6160 University Drive South, Omaha, NE 68182 USA

**Keywords:** Biomechanics, Balance, Circadian rhythms, Aging, Chronobiology

## Abstract

Human movement variability reflects the adaptive capacity of the nervous system, yet how it is influenced by aging and circadian rhythms remain unclear. Therefore, the purpose of this study was to investigate postural sway variability as a function of aging and time of day. Nineteen young and nineteen older adults completed one 60-s quite stance trial with eyes open while standing on a force platform, at 12 p.m. and 12 a.m. Postural sway variability was evaluated regarding both its magnitude (total travel distance and interquartile range) and the complexity (*a* exponent using Detrended Fluctuation Analysis) of its temporal structure using the center of pressure time series. A two-way ANOVA (2 age groups × 2 times of day) was used. Correlation analysis was also performed to further investigate the relationship between circadian regulation and postural sway complexity. Complexity was higher for the young compared to the older group independently of the time of day. Furthermore, young adults presented higher values during the morning as compared to evening, while older adults did not reveal significant differences within the day. Finally, a strong correlation was found but only for young adults. In general, our results suggested that complexity of postural sway variability is affected both by age and time of day. Aging impacts postural control by reducing the complexity of sway variability and diminishing its sensitivity to circadian influences. Future work will address the effect of chronotype, sleep, and arousal levels on these novel findings and assess their impact on overall health.

## Introduction

Many human biological processes display behavioral variations according to the time of day that the behaviors are performed. Such variations are regulated by the circadian rhythms that are approximately 24 h in duration, although other factors such as fatigue, arousal levels, and task-related constraints may also contribute to fluctuations in motor behavior (Beerendonk et al. 2024; Galy et al. 2008). Circadian regulation is driven by melatonin levels that depend on a variety of environmental conditions (Serin and Acar Tek 2019; Zisapel 2018). Disruption of this mechanism is associated with numerous physiological (Morris et al. 2016; Sellix 2016) and psychological (Hasler et al. 2010) conditions. Furthermore, the variations that occur due to the circadian rhythms may differ across groups (e.g., young versus old). The response to circadian rhythmicity is called chronotype and it is what defines a person as morning, evening, or neither (Adan et al. 2012).

Several aspects of human movement have also presented performance variations due to different times of day and different chronotypes. With respect to gait, mediolateral postural sway amplitude during gait initiation was found to be lower in the afternoon compared to the morning for Parkinson’s disease patients (Stewart et al. 2018). On the contrary, the coefficient of variation of several spatiotemporal gait parameters was found to be increased in the afternoon for people with high cutaneous sensitivity and was unaffected by the time of day for people with low cutaneous sensitivity (Korchi et al. 2019). Vaz et al. (2023) studied the effect of chronotypes and time of day on stride-to-stride fluctuations during gait which are known to exhibit a fractal-like structure. They found that 12 p.m. exhibits a higher fractal scaling exponent compared to 8 a.m. and evening-types exhibit higher fractal scaling compared to morning-types, suggesting greater temporal organization in gait variability during midday and in individuals whose biological rhythms favor later activity. These findings support the idea that circadian phase and chronotype modulate the structure of movement variability, which may extend beyond gait to other postural tasks. During standing, postural control in terms of sway has also been studied in young adults as a function of the time of day. However, this work produced contradictory results requiring further investigation. Some studies reported decreased postural sway velocity (Gribble et al. 2007) and magnitude (Forsaman et al. 2007) in the morning hours, while another study found such results to be present in the evening hours (Kwon et al. 2014). In addition, other studies have found no effect of the time of day on postural sway (Avni et al. 2006; Deschamps et al. 2013; Kohen-Raz et al. 1996; Nakano et al. 2001). These inconsistent findings highlight that time-of-day effects on balance control remain poorly understood, despite their potential relevance for fall risk, motor performance, and clinical assessment.

Regarding the effect of aging on circadian rhythms, chronotype changes have been observed in older adults and showed earlier sleep wake cycles (Duffy et al. 2015). Daily variations in cognitive function have also been observed with aging. Younger people perform better later in the day (Allen et al. 2008), while as age progresses a shift towards morningness occurs with older adults functioning better earlier in the day (Hasher et al. 2002). It has also been reported that in older adults, circadian rhythms present reduced fluctuations of physiological parameters during the day (Duffy et al. 2015; Kondratova and Kondratov 2012; van Someren 2000). The interaction between aging and time of day regarding their effect on human movement and specifically on postural sway has, however, received limited attention. This is particularly important considering the increased incidence of falls in the elderly and their relationship with postural control (Fernie et al. 1982; Johansson et al. 2017). In a recent study where such comparisons were made, the authors found that young adults exhibit a decreased magnitude of postural sway at 12 p.m. as compared to 12 a.m., while no such time-of-day effects were found in older adults (Mylonas et al. 2023). However, this study did not report how postural sway variability changes over time or in other words how time of day affects the temporal structure of postural sway variability. Such analysis of variability has been used increasingly to describe complex conditions in which linear techniques (e.g., standard deviation) have been inadequate, confounding scientific study and the development of meaningful therapeutic options (Cavanaugh et al. 2010; Goldstein et al. 1998; Toweill and Goldstein 1998). Regarding postural control, such analysis has been able to provide invaluable information for a variety of populations and under different conditions (Kelty-Stephen et al. 2024; Kyvelidou et al. 2013; Mangalam et al. 2024; Negahban et al. 2016). We expect similar insights regarding our question, namely how aging and time of day interact with respect to postural control.

Another benefit of this analytical approach is that it has allowed the development of interesting theoretical models. In particular, it has been suggested that the naturally occurring fluctuations across multiple repetitions of a motor task, such as those present in postural sway, are characterized by an optimal state of variability (Harrison and Stergiou 2015; Stergiou and Decker 2011; Stergiou et al. 2006). The capacity of an organism to operate and adapt to the demands of daily living is better enabled when the temporal structure of movement variability is “optimal”, or it has a richness of physiological complexity. This latest notion relates to the complex interactions across several control systems, feedback loops, and regulatory mechanisms, and are described with the presence of a fractal-based structure in human movement (Cavanaugh et al. 2017; Goldberger and West 1987; Harrison and Stergiou 2015; Lipsitz and Goldberger 1992; Stergiou and Decker 2011). The loss of this physiological complexity with age and disease lowers an individual's capacity for adaptation. Thus, physiological complexity is proposed to be an inherent characteristic of healthy biological systems. An excessively periodic, confined system or an excessively random, incoherent system, are both examples of a loss of this physiological complexity. In both, the fractal-based structure that characterizes healthy systems deteriorates.

As implied above, the utilization of the above theoretical model in human movement requires specific metrics to describe physiological complexity in the temporal structure of human movement variability and thus to test the model’s premises. This has been accomplished with fractal-based metrics. A geometric object having "self-similarity" across several measurement scales is the traditional definition of a fractal, which was first stated by Mandelbrot (1982). In healthy systems, motor output often follows such fractal patterns. This is reflected in a power-law relationship, where slower changes (lower frequencies) tend to have larger amplitudes (Cavanaugh et al. 2017; Harrison and Stergiou 2015). This power-law relation can be expressed as 1/f, and is referred to as pink noise, where oscillations appear self-similar when observed over seconds, minutes, hours, or days (Diniz et al. 2011). Hausdorff et al. (1996, 1998) provided early evidence for the loss of physiological complexity with aging and disease in gait variability. Aging, specifically, demonstrates a breakdown of this 1/f scaling; in other words, a loss of complexity where their movement dynamics are too random (white noise; Almurad et al. 2018; Buzzi et al. 2003; Dossey 2010; Hausdorff et al. 1997; Stergiou and Decker 2011; Vaz et al. 2020).

While the relationships between fractal patterns of locomotor activity and health are indeed intriguing, Hu et al. (2007, 2008) identified a possible neural site that is responsible for scale-invariant regulation of a neurophysiological system over a range of time scales. They demonstrated that lesioning the suprachiasmatic nucleus (SCN) of the anterior hypothalamus in rats, which is the neural node responsible for circadian rhythms, led to the disappearance of fractal patterns in both heart rate and locomotor rhythms. In addition, it is accepted that the age-related attenuation of the central timing signal generated by the SCN is associated with several health problems such as metabolic syndrome, neurodegenerative disorders, and cardiovascular diseases (Kondratova and Kondratov 2012). Previous studies suggest that some, but not all peripheral circadian oscillators exhibit age-related changes in rhythmicity (Yamazaki et al. 2002) and that some of the related tissues retain the capacity to oscillate but are not appropriately driven in vivo by physical activity rhythms (Asai et al. 2001). Information on the precise role that circadian abnormalities play in the aging process is somewhat limited, however it has been hypothesized that the fragmentation of behavioural activity with aging may worsen the age-related defects in the central clock function (Farajnia et al. 2014). Together, these considerations suggest that interventions to regulate circadian activity rhythm abnormalities are warranted in older adults (Tranah et al. 2011). Such therapeutic interventions that boost the circadian signal could arise through the restoration of the temporal structure of variability to its complex patterns, thus acting to ameliorate some of the decline seen in aged individuals.

In the present study we sought to make a first step towards the eventual development of such interventions by investigating how time of day and age affect physiological complexity in the temporal structure of postural sway variability. Based on the above presented literature, we made the following hypotheses. We hypothesized that the magnitude of postural sway variability will present higher values for older adults as compared to young adults. We also hypothesize that young adults’ sway will present higher complexity as compared to the older adults. In addition, we expect that the influence of time of day will be more apparent for young compared to older adults. Furthermore, we expect young adults to present higher complexity and lower magnitude in their postural sway during the night compared to the day, while older adults will present lower magnitude and higher complexity in their postural sway during the day compared to the night. Lastly, we hypothesized that complexity of postural sway variability will be positively correlated with circadian regulation, as imprinted in the variations of postural sway complexity within the day.

## Materials and methods

This study is based on a secondary analysis of data previously published (Mylonas et al. 2023). A brief description follows:

### Participants and apparatus

Thirty-eight healthy adults, 19 young (22.63 ± 3.43 years old with a BMI of 25.16 ± 3.79) and 19 older (60.21 ± 3.67 years old with a BMI of 29.83 ± 3.69), voluntarily participated in the study. All participants had not been taking any medication at the time or at least a month before the measurement nor had they had any orthopedic surgery in their life. None of the participants were diagnosed with any kind of sleep disorder. They all agreed to the experimental conditions and signed a consent form before the measurements. They completed the tests twice in 24 h, once at midday and once at midnight. The study was approved by the university ethics committee (approval number: EC-42/2021).

A squared (1 by 1 m) custom-made force-platform with one-dimensional force sensors in each corner was used for assessing postural sway. The sampling frequency was set at 50 Hz. To verify that our results were not dependent on the sampling frequency used, raw data were downsampled to 10 Hz and analysis processes were repeated. Results yielded from the down-sampled data were not different from the ones yielded from the original 50 Hz sampled dataset. Thus, we proceeded to use the original data for further analysis.

### Data collection and processing

Each participant completed two trials of quite standing for 1 min each with open eyes. Trials were conducted twice in a 24-h span, one at 12:00 h ± 30 min (day) and one at 00:00 h ± 30 min (night). We selected these two extreme times to be certain that if there is an effect from the independent variable, then it will be found in our experiment. Seventeen of the 38 participants completed the day collection first, while the remaining 21 completed the night collection first. Participants were asked to abstain from coffee and alcohol consumption as well as any form of intense physical activity for at least 12 h before each trial.

Raw ground reaction force data were collected and filtered using 2nd order dual band low pass Butterworth with a cutoff frequency ranging from 8 to 10 Hz according to the sum of residuals (Winter 2009). Then, the Center of Pressure (CoP) coordinates were computed for both the mediolateral and the anteroposterior axes from the filtered data.

### Present study analysis

The total travel distance of the CoP for each trial was measured to quantify the magnitude of postural sway variability. In addition, the magnitude of the CoP displacement was quantified using the Interquartile Range (IQR) for the AP anterior–posterior (IQR_AP_) and the mediolateral (IQR_ML_) axes.

Complexity of the temporal structure of the postural sway variability was quantified with the fractal-scaling exponent, *a*, which was calculated using the Detrended Fluctuation Analysis (DFA) algorithm (Peng et al. 1994) separately for the anterior–posterior (DFA_AP_) and the mediolateral (DFA_ML_) axes. Briefly this procedure is as follows. The time series is integrated and divided into windows with *n* size. Then the data from each window are fitted in a least square fit line; integrated data are then detrended by subtracting them from the fit line. The root mean square is calculated for each window and summed across the whole time series, F(n). This process is repeated for other windows with smaller and smaller *n* sizes. After the iteration of this process for all window sizes the log(F(n)) is plotted against the log(n) and a linear fit is applied. The slope of the linear fit is the fractal-scaling exponent, *a.* The range of time scales (window sizes) that was selected for this analysis was 4 to N/4, where N is the total samples of the time series (Peng et al. 1994). The results yielded from this analysis were replicated using other time scale ranges as well (e.g., 4 to N/14).

Statistical analyses were performed using SPSS (26.0 IBM statistical software). A two-way (2 × 2) repeated measures ANOVA was used (2 age groups × 2 times of day). Mauchly’s Test of Sphericity was used to test the assumption of equal variance. Bonferroni post hoc analysis was used when interactions were found significant. Effect sizes were calculated as partial eta squared $\left( {\eta_{p}^{2} } \right)$. To further investigate the connection between circadian regulation and the temporal structure of postural sway variability, correlation analysis was conducted among the absolute difference of *a* exponent between the two repeated measurements (day and night) and the highest *a* exponent value of each participant. The Pearson’s Correlation coefficient (r) was computed between the higher *a* exponent (day or night) and the inter-day absolute difference of the *a* exponent. The correlation coefficient values are interpreted as small (0.1 < r < 0.3), moderate (0.3 < r < 0.5), and strong (0.5 < r < 1.0) according to Cohen (Cohen 2013). The significance level was set to *0.05*.

## Results

### Magnitude of postural sway variability (total travel distance)

Regarding the total travel distance, no significant main effects of time (*F* = 1.998, *p* = 0.166 *η*_*p*_^*2*^ = 0.053) or group (*F* = 2.101, *p* = 0.156 *η*_*p*_^*2*^ = 0.055), nor interaction (*F* = 0.006, *p* = 0.940 *η*_*p*_^*2*^ = 0.000) between the two factors were noticed (Table 1).

**Table 1:** Total travel distance, IQR, and *a* exponent values across both times of day and age groups

		Day	Night
Travel distance (mm)	Young	796.78 (± 243.81)	758.72 (± 159.55)
	Older	925.35 (± 366.71)	882.84 (± 317.18)
IQR_AP_ (mm)	Young	10.48 (± 5.66)	9.82 (± 4.69)
	Older	8.60 (± 3.88)	8.06 (± 3.35)
IQR_ML_ (mm)	Young	7.49 (± 3.36)	9.25 (± 7.58)
	Older	7.08 (± 3.08)	6.20 (± 1.96)
*a* exponent (DFA_AP_)	Young	1.40 (± 0.11)	1.32 (± 0.07)
	Older	1.31 (± 0.12)	1.33 (± 0.11)
*a* exponent (DFA_ML_)	Young	1.25 (± 0.13)	1.29 (± 0.13)
	Older	1.20 (± 0.12)	1.18 (± 0.08)

Data are presented as group means ± SD

### Magnitude of postural sway displacement (IQR)

Regarding the IQR_AP_ and IQR_ML_ variables, no significant main effects of time (IQR_AP_:* F* = 0.545, *p* = 0.465, *η*_*p*_^*2*^ = 0.015; IQR_ML_: *F* = 0.213, *p* = 0.647, *η*_*p*_^*2*^ = 0.006) or group (IQR_AP_:* F* = 2.288, *p* = 0.139, *η*_*p*_^*2*^ = 0.060; IQR_ML_:* F* = 2.204, *p* = 0.130, *η*_*p*_^*2*^ = 0.063), nor interaction between the two factors were noticed (IQR_AP_:* F* = 0.006, *p* = 0.938, *η*_*p*_^*2*^ = 0.000; IQR_ML_:* F* = 1.916, *p* = 0.175, *η*_*p*_^*2*^ = 0.051).

### Complexity of the temporal structure of postural sway variability (a exponent)

**Fig. 1:**
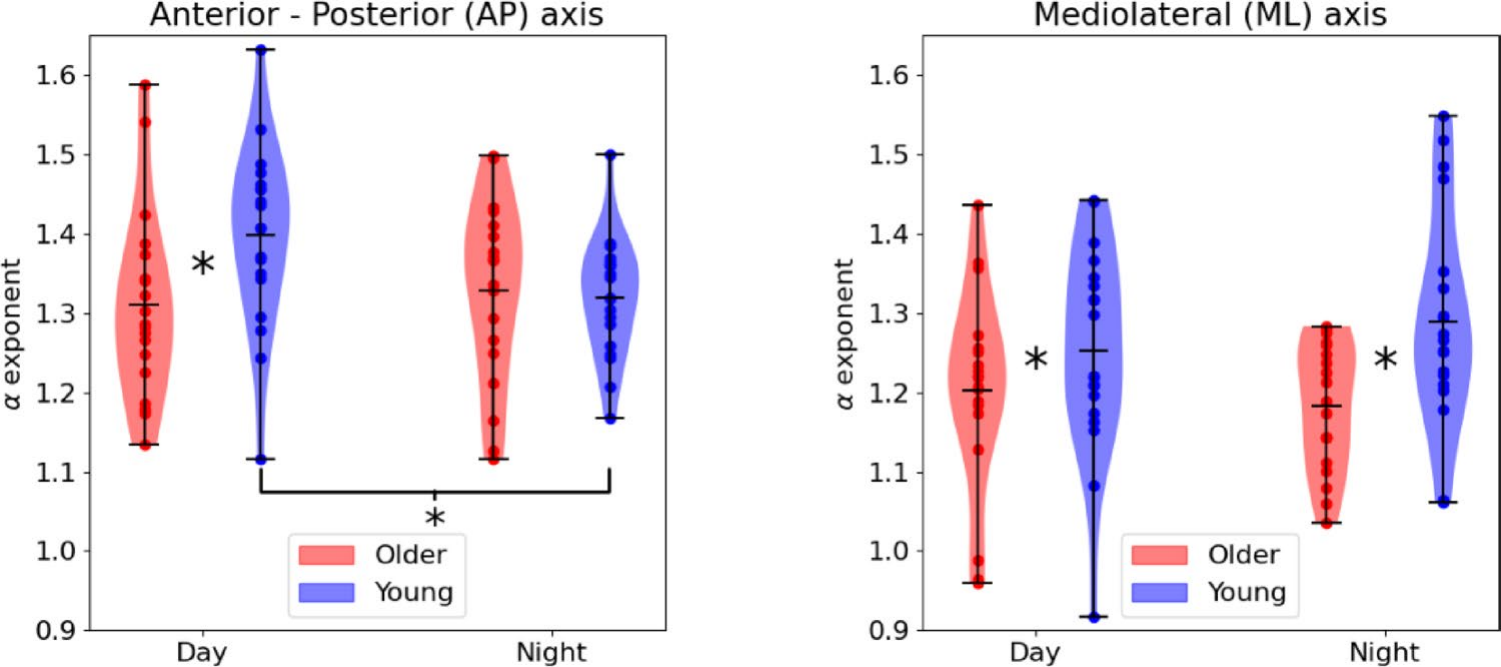
Violin plots regarding the *a* exponent for the day and night trials for: **a** DFA_AP_, and **b** DFA_ML_

Regarding the complexity of the temporal structure of postural away variability, no significant main effects of time or group were noticed for DFA_AP_. However, a significant interaction (time × group) was observed (*F* = 4.355, *p* = 0.044, *η*_*p*_^*2*^ = 0.108). Post hoc tests indicated that the young group had significantly larger *a* exponent values compared to the older group only during the day measurement (*p* = 0.025) and that the young group had significantly larger* a* exponent values during the day compared to the night measurement (*p* = 0.005; Fig. 1a).

A main effect of group was noticed for DFA_ML_ (*F* = 6.878, *p* = 0.013, *η*_*p*_^*2*^ = 0.160) where the young group had significantly larger *a* exponent values compared to the older group (Fig. 1b). No effect of time of day or interaction was observed for DFA_ML_.

**Fig. 2:**
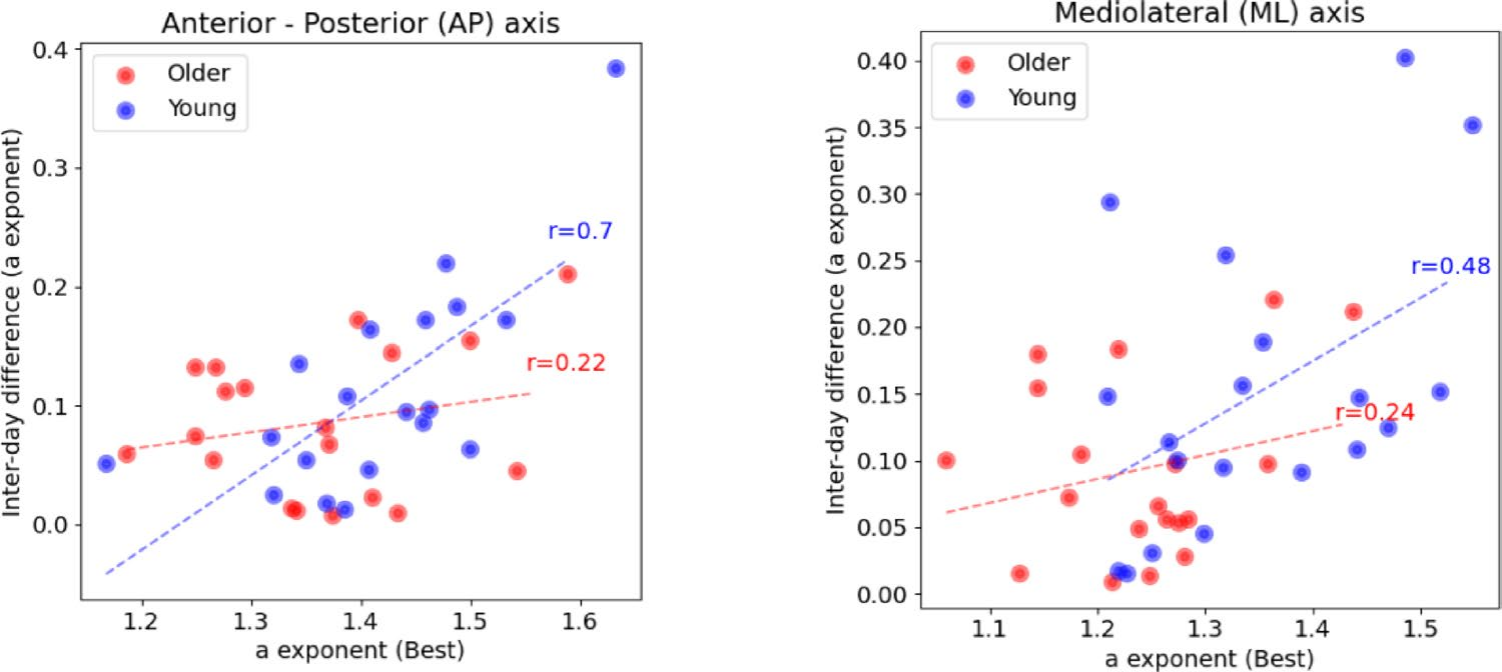
Correlations between the inter-day absolute difference in the *a* exponent and the higher *a* exponent (day or night) for: **a** DFA_AP_, and **b** DFA_ML_

Moderate to strong positive correlations were observed between the higher *a* exponent (day or night) and the inter-day difference of the *a* exponent for young adults in the AP (r = 0.7) and ML (r = 0.48) axes. Correlation coefficients for the older adults were found to be small both in the AP (r = 0.22) and the ML (r = 0.24) axes (Fig. 2).

## Discussion

The present study investigated how time of day and age affect postural sway variability. Regarding our first hypothesis, magnitude of postural sway, as measured with total travel distance and IQR, failed to unveil any differences across groups and time of day. However, our results supported the hypothesis that young adults will present higher complexity. In addition, young adults presented higher complexity during day compared to night, while older adults had no significant changes within the day. Also, the correlation between postural sway complexity and circadian regulation was found to be strong only in young adults.

We should notice that the measures of postural sway magnitude (total travel distance and IQR) were lower during night. Young adults also presented non-significant lower magnitude of postural sway compared to the older adults. These results agree with previous findings, where they have found that older adults had larger values for the magnitude of postural sway compared to young (Chen and Qu 2019; Viswanathan and Sudarsky 2012), possibly because of the degeneration of sensory systems (Martens and Almeida 2012), CNS (Bergström 1973; Richter 1980) and the muscular system (Larsson et al. 2019; Roubenoff 2000), and therefore diminished ability to control posture. However, our results could not statistically verify these previous literature findings. It is possible that the absence of statistical significance regarding the magnitude of postural sway variability in our data was due to the relatively “young” age of our older adult sample (a group mean of 60.21 years old). On the other hand, our results also verified previous literature observations that such linear metrics are inadequate to fully study variability in human performance (Hausdorff et al. 1995, 1996; Stergiou and Decker 2011).

In terms of complexity of the temporal structure of postural sway variability, differences between the two age groups were present in the *a* exponent values. Our values are also similar with ones previously reported for both young and older adults (da Costa Barbosa and Vieira 2017; Duarte and Sternad 2008). Young adults had significantly larger values compared to older adults, indicating stronger presence of 1/f fractal dynamics in their CoP movement (Lin et al. 2008; Riley et al. 2012; van den Hoorn et al. 2018). These results agree with the notion of loss of complexity in older populations leading to breakdown of fractal properties in the temporal structure of movement variability (Cavanaugh et al. 2017; Stergiou and Decker 2011). In postural control, such patterns may reflect the efficiency of balance mechanisms. There is compelling evidence suggesting that the complexity of postural sway provides a rich source of information that could be relevant to the diagnosis and management of a variety of diseases that affect an aging population (Kelty-Stephen et al. 2024; Kyvelidou et al. 2013; Mangalam et al. 2024; Stergiou and Decker 2011). Similar alterations to postural sway variability into more random structure has also been linked to fall risk in older adults, highlighting its potential clinical relevance (Zhou et al. 2017).

Our third and fourth hypotheses were partially supported, as an effect of time on postural sway complexity was observed only for young adults. This suggests that the influence of time of day was apparent in young adults, while in older adults did not matter. However, our predictions about what time of day we are going to observe higher postural sway complexity were not confirmed, as young adults presented higher *a* exponent values during the day. Nevertheless, this result is not that strong as the interaction of age with time of day yield a medium effect size. Therefore, we recommend that further experimentation is required to draw definitive and reliable conclusions. The characterization of a specific group of individuals as morning or evening types should be accompanied by biochemical markers (e.g. melatonin) and questionnaires (e.g., Morningness–Eveningness Questionnaire; Horne and Ostberg 1976), and not be hypothesized solely based on the age. Despite that, the within day differences found in young adults can be interpreted as a healthy functioning SCN. In older adults, the lack of any such differences could be the result of both changes in complexity (Stergiou and Decker 2011) and disrupted circadian regulation (Duffy et al. 2015; Kondratova and Kondratov 2012; van Someren 2000), as consequence of decreased neural activity in the SCN (Swaab et al. 1985). This is also supported by our results where the older group presented lower postural sway complexity, in addition to the non-significant effect of time of day.

Finally, the strong correlation that was noticed for the young adults further supports the hypothesis that circadian regulation and complex patterns in the variability of human physiology are related. Our results suggest that the more complexity the postural sway presents, the higher are the differences due to the time of day. The fact that this result was presented only in young adults may indicate that in older adults, movement complexity may become independent from circadian regulation possibly due to SCN activity deterioration, as mentioned above.

The results of this experiment highlight that complexity is dependent on both age and time of day. Changes in complexity in human movement variability is a common consequence of aging (Stergiou and Decker 2011), while other research results have pointed to a relationship between circadian rhythms and temporal structure of movement variability, as it is believed to be regulated by the same brain center, the SCN (Hu et al. 2013). Recent literature also suggests that complexity can be restored in older adults and pathological populations (Hunt et al. 2014; Likens et al. 2021; Vaz et al. 2020). Such interventions can benefit from our results by attempting to restore movement complexity at the time of day where complexity is at the lower level. However, this claim may be invalid if the outcome of these interventions is not dependent in the time of day. Future research that will attempt to restore movement complexity in different times of the day is necessary to support or exclude this hypothesis. Additionally, it is noteworthy that the movement complexity and the regulation of circadian rhythms not only exhibit a mutual relationship, but treatment targeting the SCN also appears to exert an influence on fractal patterns. Transplantation of a ‘young’ SCN into aged animals resulted in improvements in numerous rhythmic functions, including behavioural rhythms in locomotion (Li and Satinoff 1995). It is however unknown if circadian deficits may also be restored along with movement complexity. Future research should address the coupling of circadian biorhythm with movement complexity to further clarify the relationship between the two.

Our results should be considered in lieu of the following limitations which we plan to address in future experiments. First, our experimental protocol is comprised of only two extreme measurement points within the day. Our findings could be strengthened by using a protocol with more measurements during the 24-h span and more repeated trials per measurements. Additionally, the older group in our experiment consisted of relatively “young” individuals (60.21 years old). Future research should aim on older individuals in whom shift towards morningness, and complexity decreases have been pre-determined and quantified to better construct experimental groups. Finally, our results do not allow for an accurate estimation of the relationship between circadian regulation and movement complexity but rather describe the effect of time of day. To establish this relationship, future research should account for the chronotype and sleep schedule of the participants as well as factors such as sleepiness, alertness and levels of arousal. Identification of the chronotype of participants using questionnaires should be included to verify the shift towards morningness.

We have provided preliminary evidence that complexity in the temporal structure of postural sway variability could be affected by the interaction of aging and time of day. This study is a first step on our efforts to identify if we can develop therapeutic interventions that can boost the circadian signal through the restoration of the temporal structure of movement variability to its complex patterns, thus acting to ameliorate some of the decline seen in aged individuals.

## Data Availability

All data supporting the findings of this study are provided within the paper. Raw time series data can be obtained from the corresponding author upon request.
