# Correction: Postural sway variability in young adults presents higher complexity during morning compared to evening hours while in older adults remains the same

**DOI:** 10.1007/s00221-025-07159-9

**Published:** 2025-09-18

**Authors:** Vasileios Mylonas, Stylianos Grigoriadis, Christos Chalitsios, Nick Stergiou, Thomas Nikodelis

**Affiliations:** 1 AUTH Biomechanics, Department of Physical Education, & Sport Science, Aristotle University, 570 01 Thessaloniki, Greece; 2 Division of Biomechanics and Research Development, Department of Biomechanics and Center for Research in Human Movement Variability, University of Nebraska at Omaha, 6160 University Drive South, Omaha, NE 68182 USA

**Correction to: Experimental Brain Research (2025) 243:176**. 10.1007/s00221-025-07121-9

In the original version of the article, the following co-author name has been incorrectly published.

The Incorrect name: Stylianos Grioriadis.

The correct name: Stylianos Grigoriadis.

The original article has been updated.

